# Determinants of Willingness to Pay for Health Insurance in Germany—Results of the Population-Based Health Study of the Leipzig Research Centre for Civilization Diseases (LIFE-Adult-Study)

**DOI:** 10.3389/fpubh.2020.00456

**Published:** 2020-08-28

**Authors:** André Hajek, Cornelia Enzenbach, Katarina Stengler, Heide Glaesmer, Andreas Hinz, Susanne Röhr, Janine Stein, Steffi G. Riedel-Heller, Hans-Helmut König

**Affiliations:** ^1^Department of Health Economics and Health Services Research, Hamburg Center for Health Economics, University Medical Center Hamburg-Eppendorf, Hamburg, Germany; ^2^Institute for Medical Informatics, Statistics and Epidemiology, University of Leipzig, Leipzig, Germany; ^3^Department of Psychiatry, Psychosomatic Medicine and Psychotherapy, Helios Park Hospital Leipzig, Leipzig, Germany; ^4^Department of Medical Psychology and Sociology, University of Leipzig, Leipzig, Germany; ^5^Institute of Social Medicine, Occupational Health and Public Health, University of Leipzig, Leipzig, Germany

**Keywords:** health insurance, willingness to pay, personality, big five, LIFE-Adult-Study

## Abstract

**Objective:** To investigate which factors are associated with the willingness to pay (WTP) for health insurance.

**Methods:** The analysis (*n* = 1,248 individuals) is based on data of a large population-based study—the Health Study of the Leipzig Research Centre for Civilization Diseases (LIFE-Adult-Study). With regard to WTP for health insurance, a contingent valuation method with a payment card was used. Several explanatory variables were included. For example, personality factors (in terms of agreeableness, conscientiousness, extraversion, neuroticism, and openness to experience) were assessed using the NEO-16 Adjective Measure.

**Results:** Average WTP for health insurance per month equaled about €240 which corresponds to ~14% of household net equivalent income. Multiple regressions showed that an increased WTP was associated with lower age (β = −1.7, *p* < 0.001), higher (log) household net equivalent income (β = 153.6, *p* < 0.001), higher social support (β = 2.0, *p* < 0.05), and private health insurance (β = 131.1, *p* < 0.001). Furthermore, an increased WTP for health insurance was associated with higher openness to experience (β = 10.1, *p* < 0.05), whereas it was not associated with agreeableness, conscientiousness, extraversion, and neuroticism.

**Conclusion:** The quite large amount of average WTP for health insurance may suggest that individuals accept current contributions to health insurances and would probably accept higher contributions. While previous studies mainly focused on individuals in late life, we identified a link between socioeconomic, health-related factors, and personality factors (in terms of openness to experience) and WTP in the general adult population.

## Introduction

Like other industrialized countries, Germany is expected to change its demographic structure. For example, the proportion of individuals ≥65 years is projected to increase to about 34% in 40 years (1). These demographic shifts are accompanied by great challenges to the health care system in Germany. Thus, it is important to preserve the support for the solidary system of health insurance in the upcoming decades. For this reason, it is of utmost importance to identify the preferences for health insurance in Germany. Based on a review of electronic databases (Medline, PsycINFO, CINAHL) and a manual search of relevant studies (reference lists of the included studies), two reviewers only identified some comparable studies (2–9). More precisely, most of the current knowledge stems from developing countries which do not have comprehensive health insurance systems (2–4, 7–9).

Thus far, only a few studies exist examining the determinants of willingness to pay (WTP) for health insurance. More precisely, it has for example been shown that WTP for health insurance is associated with higher income, being male, higher educational level and higher individual health care costs among individuals in old age in Germany (5, 6). However, to date it remains largely unknown which factors are associated with the WTP for health insurance in the general adult population. Consequently, the aim of this study was to investigate which factors are associated with the WTP for health insurance in the adult population in Germany.

Some key characteristics of the health care system in Germany are worth noting. While ~90% of the population is insured by social statutory health insurance (SHI), about 10% is insured by private health insurance (PHI). Irrespective of general health, contributions for SHI depend on the income. While the vast majority cannot opt for a PHI, particularly self-employed individuals and employees who exceed a certain income-threshold can opt for a PHI and keep this membership even after retirement. In contrast to SHI, premiums of PHI largely depend on health status when entering the PHI (10).

For example, in 2009 the average contribution per SHI member was about €280 (nearly 15% of gross income) per month in Germany (11). Members' spouses or children (≤25 years) without an income are also covered by this contribution. The contributions are equally financed by employees and employers. In the year 2020, this equals 14.6% of income subject to contributions (plus an additional contribution to health insurance which is equally financed; the average additional contribution is 1.1% in the year 2020).

Both PHI and SHI offer coverage of most health care expenses (inpatient and outpatient treatment). Only small co-payments exist for these services in the SHI. In the PHI, the amount of deductibles depend on the individual contract signed. In Germany, members of the SHI do not receive invoices from the health providers (principle of benefits in kind). Instead, they show their membership card when using health services. Thus, the providers are directly paid by the SHI, whereas PHI members receive invoices from providers first. After payment, they receive a reimbursement from the PHI.

In case of PHI, the basis for payment between service providers and health insurance companies is the “scale of fees for doctors” (in German: “Gebührenordnung für Ärzte”), whereas the “uniform valuation standard” (in German: “Einheitlicher Bewertungsmaßstab”) regulating the accounting of outpatient health services within the SHI. The private health insurance companies have to deal without state subsidies—which is in contrast to the statutory health insurance. Due to the medical-technical progress and the increased demand for health care services, premiums markedly increased in the past.

## Methods

Data are derived from the Health Study of the Leipzig Research Centre for Civilization Diseases (LIFE-Adult-Study). This is a large population-based study of a representative sample of the inhabitants of the city of Leipzig (Germany). The study is conducted by the Leipzig Research Centre for Civilization Diseases (LIFE). From the population registry office, a random sample stratified by age (from 40 to 80 years) and sex was drawn. From 2011 to 2014, 10,000 randomly selected inhabitants of Leipzig completed the baseline examination. Only one exclusion criterion was present (not being pregnant). The response rate for the LIFE-Adult-Study was 33%.

Written informed consent was given by the participants at the study center. Afterwards, a set of instruments was used covering structured interviews (sociodemographic data, medical history, medications, as well as lifestyle-related items) as well as medical examinations like blood samples, or cognitive functioning. The participants received a small financial incentive (€20). In total, 1,430 individuals answered the questions about the WTP for health insurance. In our resulting analytical sample, n equaled 1,248 individuals due to some missing variables in the independent variables. Further details are given elsewhere (12). The study was approved by the ethics committee of the University of Leipzig.

### Outcome Measure: Willingness to Pay for Health Insurance

A contingent valuation technique was used to measure participants WTP for health insurance (13–17). A payment card was used (15), introduced as follows: “Imagine, you had no health insurance: In consideration of your monthly household net income, how much would you maximally be willing to pay per month for health insurance, if it provides the same standards as your current health insurance?” The payment card offered nine different amounts of money as answer possibilities (€50, €100, €200, €300, €400, €500, €700, €1,000, > €1,000). Participants were asked to report the amount they would be willing to pay in the first column. In addition, they were asked to state the amount they would definitely not be willing to pay.

Following Bock et al. (5), the mid-point technique was used in the present study which means that the mid-point of the interval defined by the amount of the maximum WTP and the minimum an individual would not pay (values of > €1,000 were transformed to €1,250) was used to compute the outcome measure.

### Independent Variables

Based on some recent studies and theoretical considerations (5, 6), we included the following independent variables: gender, age, log household net equivalent income (in Euro), and health insurance (statutory health insurance; private health insurance). Furthermore, the 6-item version of the Lubben Social Network Scale (18) (LSNS-6) was used to quantify social contacts/social support. This scale ranges from 0 to 30, with higher values reflecting larger social networks as well as more social support. Good psychometric properties of the scale have been shown (18). To quantify depressive symptoms, the Center for Epidemiological Studies Depression (CES-D) was used (19), consisting of 20 items (in each case: 0 = rarely/almost none of the time to 3 = most or all of the time). The score ranges from 0 to 60 (higher values reflecting more depressive symptoms).

The NEO-16 Adjective Measure (20) was used to quantify five personality factors. On a 7-point scale (from 1 = strongly disagree to 7 = strongly agree), items were rated. The common introduction was “I see myself as …” For example, compared with the Ten Item Personality Inventory, it showed better reliability (20). Furthermore, it fitted the Big-Five factor structure and can serve as reasonable proxy for longer Big-Five measures (20).

It is worth noting that, most commonly, personality is divided into five big traits (21): Agreeableness, Conscientiousness, Extraversion, Neuroticism, and Openness to Experience. Agreeableness refers to the tendency to be trusting and cooperative. Conscientiousness refers to the tendency to be reliable and careful. Extraversion refers to the tendency to be outgoing, sociable, dominant, and active. Neuroticism refers to the tendency to experience negative emotional states like anger, depression, or anxiety. Openness to experience refers to the tendency to seek out novelty and to be creative. We think that it is worth including these personality characteristics because it has been shown in another setting that personality characteristics are associated with the willingness to pay for market goods (bottles of wine) (22). Moreover, it has been demonstrated that personality factors are important for health care use (23, 24). Thus, it appears plausible that personality is associated with the willingness to pay for health insurance in Germany.

### Statistical Analysis

First, sample characteristics were displayed. Second, to estimate the determinants of WTP, multiple linear regressions were conducted. The level of significance was fixed at 5%. Stata 16.0 (Stata Corp., College Station, Texas) was used to perform statistical analysis.

## Results

### Sample Characteristics

Sample characteristics for our analytical sample (*n* = 1,248 individuals) are depicted in Table 1. In total, 50.3% of the individuals were female and mean age equaled 56.2 years (SD: 11.7 years). The mean WTP for health insurance per month was ~€239.2 (SD: €178.5). Further details are displayed in Table 1.

**Table 1:** Sample characteristics for the analytical sample (*n* = 1,248).

**Variables**	**Mean (SD)/*N* (%)**
Sex: *N* (%)	
Men	620 (49.7%)
Women	628 (50.3%)
Age: Mean (SD)	56.2 (SD: 11.7)
Health insurance: *N* (%)	
Statutory health insurance	1,129 (90.5%)
Private health insurance	119 (9.5%)
Household net equivalent income in Euro: Mean (SD)	1,718.9 (SD: 903.0)
Social support (Lubben Social Network Scale): Mean (SD)	16.7 (SD: 5.2)
Depressive symptoms (Center for Epidemiological Studies Depression Scale): Mean (SD)	10.3 (SD: 6.9)
Personality factors: Mean (SD)	
Agreeableness	5.9 (SD: 1.0)
Conscientiousness	5.9 (SD: 0.8)
Extraversion	3.6 (SD: 1.2)
Neuroticism	3.3 (SD: 1.1)
Openness to experience	5.4 (SD: 0.9)
Willingness to pay for health insurance in Euro: Mean (SD)	239.2 (SD: 178.5)

*N, number of observations; SD, standard deviation*.

### Regression Analysis

The White test for heteroscedasticity in the error distribution was computed. The test statistics led to the rejection of the null hypothesis of homoscedasticity (White's general test statistic equaled 116.7, *p* < 0.01). Consequently, robust standard errors were used in our study. Furthermore, the normality of residuals was checked using standardized normal probability plots. According to that, the residuals have an approximately normal distribution.

Results of multiple regression analysis are described in Table 2. *R*^2^ equaled 0.31. We tested for multicollinearity using variance inflation factors (VIF). However, VIFs were rather low (highest VIF was 1.3, mean VIF was 1.2) indicating that multicollinearity was not a threat to our findings. Regressions showed that an increased WTP was significantly associated with lower age (β = −1.7, *p* < 0.001), higher (log) income (β = 153.6, *p* < 0.001), higher social support (β = 2.0, *p* < 0.05), and private health insurance (β = 131.1, *p* < 0.001). In contrast, it was neither associated with gender nor with depressive symptoms. Furthermore, regressions showed that an increased WTP for health insurance was associated with higher openness to experience (β = 10.1, *p* < 0.05), whereas it was not associated with agreeableness, conscientiousness, extraversion, and neuroticism.

**Table 2:** Determinants of willingness to pay for health insurance.

**Independent variables**	**Willingness to pay for health insurance**
Women (Reference category: men)	−7.16
	(9.44)
Age (in years)	−1.74^***^
	(0.35)
Private health insurance (reference category: statutory health insurance)	131.10^***^
	(20.80)
Log household net equivalent income	153.55^***^
	(11.69)
Social support (Lubben Social Network Scale)	1.97^*^
	(0.91)
Depressive symptoms (Center for Epidemiological Studies Depression Scale)	−0.59
	(0.64)
Personality factors (NEO-16 Adjective Measure)	
Agreeableness	−5.95
	(5.35)
Conscientiousness	−2.07
	(5.36)
Extraversion	−0.63
	(3.94)
Neuroticism	−4.73
	(4.47)
Openness to experience	10.13^*^
	(4.67)
Constant	−809.17^***^
	(94.75)
Observations	1,248
*R*^2^	0.31

Results of multiple linear regression analysis. Beta-coefficients are reported (unstandardized); robust standard errors in parentheses;

*** *p < 0.001*,

^**^ p < 0.01,

* *p < 0.05, ^+^p < 0.10; Lubben Social Network Scale ranges from 0 to 30, with higher values reflecting larger social networks as well as more social support. The Center for Epidemiological Studies Depression ranges from 0 to 60, with higher values reflecting more depressive symptoms*.

## Discussion

Based on a large population-based study, the purpose of our study was to examine the factors associated with the willingness to pay (WTP) for health insurance. Average WTP for health insurance per month equaled about €240 which corresponds to ~14% of household net equivalent income. Multiple regressions showed that an increased WTP was associated with younger age, higher income, higher social support and private health insurance. In addition, an increased WTP for health insurance was associated with higher openness to experience, whereas it was not associated with agreeableness, conscientiousness, extraversion, and neuroticism.

Thus far, two German studies investigated the WTP for health insurance among individuals in late life (5, 6). Our findings extend these findings by determining correlates of WTP in the adult population. In total, we revealed similar findings (e.g., a link between higher income and an increase in WTP). Another benefit of the current study was to identify a link between personality factors (i.e., openness to experience) and the WTP. It appears plausible that openness to experience is associated with an increased WTP because individuals scoring high in openness to experience tend to be politically left-wing (25). In turn, left-wing individuals are generally more willing to accept higher government taxation because of their high preferences for social services, redistribution, and equality. Thus, one may conclude that individuals scoring high in openness to experience may also be more willing to accept higher premiums or deductibles for health insurance. However, future studies are needed to confirm our findings and to elucidate the underlying mechanisms.

Some strengths and limitations of this study are worth noting. A large population-based study (general adult population) was used. Adding to our current knowledge, this is the first study to examine the association between personality and willingness to pay for health insurance. This is a cross-sectional study with the widely acknowledged restrictions (e.g., with regard to intraindividual changes). In this study, the contingent valuation instrument was used. It has been discussed critically in previous research (5), particularly because it might affect the decisions (referring to selected money categories in the payment card and the fact that the participants could see all answer possibilities). Although the latter fact could be avoided by using bidding game strategies, these very time-consuming strategies are very difficult to implement in large cohort studies due to time constraints. Furthermore, some sample selection bias has been identified in the LIFE-Adult-Study (26).

## Conclusion and Future Research

The quite large amount of average WTP for health insurance may suggest that individuals accept current contributions to SHI and would probably accept higher contributions. While previous studies mainly focused on individuals in late life, we identified a link between socioeconomic, health-related factors, and personality factors (in terms of openness to experience) and WTP in the general adult population. Furthermore, future research is required to gain deeper insights into the determinants of WTP among different subgroups [e.g., stratifying between active SHI members, voluntarily insured individuals (above the mentioned income threshold), family members (who do not need to pay themselves), occupational status and so on].

## Data Availability Statement

The dataset analyzed during the current study is available from the corresponding author upon reasonable request.

## Ethics Statement

The studies involving human participants were reviewed and approved by Ethics committee of the University of Leipzig. The patients/participants provided their written informed consent to participate in this study.

## Author Contributions

AHa conceptualized and designed the study, conducted the statistical analyses, interpreted the data, and drafted the manuscript. CE, KS, HG, AHi, SR, JS, and SR-H revised the manuscript for intellectual content, read, and approved the final version of the manuscript. H-HK conceptualized and designed the study, supervised the drafting of the manuscript, supported in interpreting the data, and revised the manuscript for intellectual content. All authors read and approved the final manuscript.

## Conflict of Interest

The authors declare that the research was conducted in the absence of any commercial or financial relationships that could be construed as a potential conflict of interest.
